# Supplementary material for: Long-Term Effects of COVID-19 on Workers in Health and Social Services in Germany
Source: Int J Environ Res Public Health. 2022 Jun 7;19(12):6983. doi: 10.3390/ijerph19126983 (PMC9222999; doi:10.3390/ijerph19126983)
Supplement: Supplementary file 1 [file ijerph-19-06983-s001.zip › ijerph-1716575-supplementary.pdf]

Table S1. Symptoms of post-COVID-19 syndrome (n=1406/72.8%)

**Symptoms**		**PCS n / %**
Fatigue/ exhaustion	Mild	404 / 28.7
	Moderate	519 / 36.9
	Severe	242 / 17.2
Concentration/ memory problems	Mild	470 / 33.4
	Moderate	391 / 27.8
	Severe	133 / 9.5
Shortness of breath	Mild	384 / 27.3
	Moderate	308 / 21.9
	Severe	102 / 7.3
Headache	Mild	297 / 21.1
	Moderate	216 / 15.4
	Severe	67 / 4.8
Loss of taste/ smell	Mild	245 / 17.4
	Moderate	164 / 11.7
	Severe	126 / 9.0
Joint / limb pain	Mild	187 / 13.3
	Moderate	204 / 14.5
	Severe	77 / 5.5
Cough	Mild	207 / 10.7
	Moderate	80 / 5.7
	Severe	11 / 0.8
Rhinitis	Mild	122 / 8.7
	Moderate	43 / 3.1
	Severe	4 / 0.3
Sore throat	Mild	102 / 7.3
	Moderate	31 / 2.2
	Severe	4 / 0.3
Abdominal pain	Mild	79 / 5.6
	Moderate	36 / 2.6
	Severe	8 / 0.6
Diarrhoea	Mild	59 / 4.2
	Moderate	27 / 1.9
	Severe	8 / 0.6
Nausea/ vomiting	Mild	60 / 4.3
	Moderate	26 / 1.8
	Severe	3 / 0.2
Fever	Mild	16 / 1.1
	Moderate	9 / 0.6
	Severe	1 / 0.1
